# *CMYB1* Encoding a MYB Transcriptional Activator Is Involved in Abiotic Stress and Circadian Rhythm in Rice

**DOI:** 10.1155/2014/178038

**Published:** 2014-04-01

**Authors:** Min Duan, Peng Huang, Xi Yuan, Hui Chen, Ji Huang, Hongsheng Zhang

**Affiliations:** State Key Laboratory of Crop Genetics and Germplasm Enhancement, Nanjing Agricultural University, Nanjing 210095, China

## Abstract

Through analysis of cold-induced transcriptome, a novel gene encoding a putative MYB transcription factor was isolated and designated *Cold induced MYB 1 (CMYB1)*. Tissue-specific gene expression analysis revealed that *CMYB1* was highly expressed in rice stems and nodes. qRT-PCR assay indicated that *CMYB1* was dramatically induced by cold stress (>100-folds) and induced by exogenous ABA and osmotic stress. Interestingly, *CMYB1* showed rhythmic expression profile in rice leaves at different developmental stages. Subcellular localization assay suggested that *CMYB1*-GFP (green fluorescent protein) fusion protein was localized in the nuclei. Moreover, *CMYB1* exhibited the transcriptional activation activity when transiently expressed in rice protoplast cells. Taken together, *CMYB1* probably functions as a transcriptional activator in mediating stress and rhythm responsive gene expression in rice.

## 1. Introduction

Environmental stresses such as cold, drought, and salinity adversely affect plant growth and crop productivity. To cope with such stress, plants initiate a number of molecular, cellular, and physiological changes to respond and adapt to various stresses. The transcriptional gene regulation of stress-related genes is crucial in these responses and adaptations [1, 2]. The transcription factors play a predominant role in transcriptional regulation of stress responses in plants. Based on microarray or next-generation sequencing (NGS) technologies, many transcription factors regulated by abiotic stress were identified. However, compared with the big number of transcription factor genes present in plant genome, only a few of them have been functionally analyzed so far. The DREB/CBF transcription factors play a central role in both ABA-dependent and ABA-independent pathways via binding the C-repeat/dehydration response elements (CRT/DRE), which often exist within the promoter regions of the stress related genes [3, 4]. The DREB/CBF genes have been used in engineering tolerance against abiotic stress in crops [5–7]. Except for DREB/CBF transcription factors, some other types of transcription factors have been identified to be involved in stress signal transduction, such as bZIP transcription factors [8, 9], MYB proteins [10], NAC transcription factors [11, 12], WRKY proteins [13], and zinc finger proteins [14].

MYB transcription factors are present widely in eukaryotes [15]. MYB proteins contain one, two, or three imperfect repeats (51–53 amino acids) in their DNA-binding domain, and they are further classified into three subfamilies, type MYBR2R3, type MYBR1R2R3, and MYB-related, depending on the number of repeats in their MYB domains [16, 17]. Among them, the R2R3-type MYB proteins form a big MYB subfamily containing more than 100 members in high plants, such as* Arabidopsis* and rice. A number of MYB genes have been found to be induced by abiotic stress and their genetic engineering may improve stress tolerance in plants.* AtMYB60*, a R2R3-MYB gene of* Arabidopsis*, was the first identified transcription factor involved in the regulation of stomata movements [18].* AtMYB60 *was specifically expressed in guard cells, and its expression was negatively modulated by drought. A null mutation in* AtMYB60 *resulted in the constitutive reduction of stomata opening and decreased wilting under water stress conditions.* OsMYB3R-2 *is a MYB transcription factor induced by cold, drought, and salt stresses in rice. The* Arabidopsis *and rice transgenic plants overexpressing* OsMYB3R-2* showed increased cold tolerance [10, 19]. Further studies showed that* OsMYB3R-2* functions as a MYB transcription factor targeting* OsCycB1;1*, which was involved in the G2/M phase transition at low temperature. The transcript level of* OsCPT1*, a putative member of DREB1/CBF pathway, was also enhanced by OsMYB3R-2 [19]. Recently, another rice MYB gene* OsMYB2* was functionally studied. Expression of* OsMYB2 *was upregulated by salt, cold, and dehydration stresses. Overexpression of* OsMYB2* increased salt, cold, and dehydration tolerance. The enhanced proline and soluble sugar accumulations and activities of antioxidant enzymes, including peroxidase, superoxide dismutase, and catalase, may underlie the enhanced stress tolerance [20]. Besides in* Arabidopsis* and rice, several MYB genes from other plants, such as apple, wheat, and tobacco, also showed the roles in improving abiotic stress tolerance [21–23].

In the present study, a novel MYB transcription factor gene* CMYB1 *was identified through transcriptome analysis in rice under cold stress. Gene expression analysis suggested that* CMYB1* was largely induced upon cold stress and also induced by osmotic and drought stresses. Interestingly,* CMYB1* showed the significant rhythmic expression profile, implying that* CMYB1 *might play an important role in cold tolerance through a circadian rhythm regulation manner.

## 2. Materials and Methods

### 2.1. Plant Material and Stress Treatments

Rice cultivar Jiucaiqing (*Oryza sativa *L. subsp.* japonica*) was used in this experiment. The rice seeds were sterilized in 0.3% NaClO for 15 minutes, germinated, and cultured in Yoshida's nutrition solution in the growth chamber with a light cycle of 16 h light at 28°C and 8 h dark at 22°C until three-leaf stage. Some seedlings were transferred to the field for further growing; then the stem, node, leaf blade, leaf sheath, immature panicle, mature panicle, and anther were harvested at proper time.

For abiotic stress treatments, the three-leaf seedlings were transferred to Yoshida's nutrition solution containing 100 mM NaCl, 20% PEG 6000 (providing an osmotic potential of –0.54 MPa), and 0.1 mM ABA, respectively, or cultured in the nutrition solution under the condition of 4°C. The seedlings were sampled at different time points after each treatment, immediately frozen in liquid nitrogen, and stored at −80°C until RNA was isolated. For dehydration treatment, the seedlings were taken off from solution and kept at a natural dehydration situation for 12 h and then put back in the nutrition solution for recovery.

### 2.2. RNA Isolation and First-Strand cDNA Synthesis

The total RNA was extracted using the Trizol reagent (Invitrogen, USA) according to the manufacturer's protocol. The remaining genomic DNA in the RNA samples was removed by treating with DNase I (Promega, USA) at 37°C for 15 min. The first-strand cDNA was synthesized with 2 *μ*g of purified total RNA using the reverse transcription system (Promega, USA) according to the manufacturer's protocol.

### 2.3. Cloning of* CMYB1*

The coding sequence of* CMYB1 *was amplified from cDNA of three-leaf rice seedling by PCR using the primers as follows: forward primer: 5′-TGGGAGGAGTTCGGTTTT-3′; reverse primer: 5′-CCTCTTCTTCCCGCCTTA-3′. The PCR product was purified and cloned into pEASY-T vector (TransGen, China) and sequenced. Plasmid was isolated from single clone, which was confirmed with correct sequencing result, and used as template for next construction of expression proteins.

### 2.4. Quantitative Real-Time RT-PCR

The* CMYB1* primers (forward primer: 5′-TGCAGGCGCCAAATACTAAGATG-3′; reverse primer: 5′-CCACTACGCTCTTTCCGAATAGCC-3′) were used for quantitative real-time PCR analysis. A constitutively expressed gene* 18S rRNA *was used as an internal control [25]. The primers of* 18S rRNA* were as follows: forward primer: 5′-ATGGTGGTGACGGGTGAC-3′; reverse primer: 5′-CAGACACTAAAGCGCCCGGTA-3′.

### 2.5. Subcellular Localization of* CMYB1*::GFP Fusion Protein

To construct* CMYB1*::GFP fusion protein, the* CMYB1 *fragment was amplified using the primers as follows: forward primer: 5′-GCTCTAGAAATGGAGATGGCCTGTT-3′; reverse primer: 5′-GTTCCCGGGTGTCACAAGCAC-3′, where the underlines indicate the restriction digestion site of* Xba* I and* Sma* I, respectively. Then this fragment was cloned into the* Xba* I and* Sma* I sites of plant transient expression vector pA7 [26] to construct* CMYB1*::GFP fusion protein under the control of CaMV35S promoter. 35S::GFP fusion protein was used as control. Both of the fusion proteins were then transferred into onion epidermal cells by particle bombardment and the cells were examined with confocal laser scanning microscopy (Nikon ECLIPSE 80i, Japan).

### 2.6. Transcriptional Activity Assay in Rice Protoplast

The transcriptional activity assay of* CMYB1* was determined in rice protoplast by dual luciferase reporter assay system (Promega, USA). To construct reporter plasmids, we used the pGL3-Basic vector, in which two random* cis*-elements, 5×GAL4 and 3×DRE, and a minimal TATA promoter [27] were cloned in the MCS before luciferase reporter gene. The 35S::DBD- (GAL4-DNA binding domain-)* CMYB1* effector plasmid was constructed by inserting DNA fragments containing* CMYB1* coding region and GAL4-DNA binding domain sequence of pGBKT7 vector (Clontech, USA) into the* Spe* I and* Xba* I sites of pA7. Similarly, 35S::DBD and 35S::DBD-ZFP179 plasmids [14] were constructed as negative control and positive control, respectively. To enhance the background expression level, 35S::OsDREB1A effector plasmid that can bind to DRE* cis*-element was constructed [28]. The renilla luciferase was used as endogenous control. All the plasmids were transferred into rice protoplast by polyethylene glycol and cultivated overnight for the expression of luciferase genes. The luciferase activity was detected via GLOMAX 20/20 Luminometer (Promega, USA).

## 3. Results and Discussion

### 3.1. Sequence Analysis of* CMYB1*

Based on the cold-responsive transcriptome analysis in rice (our unpublished data), an EST probe (probe ID: Os.8149.1.S1_at) encoding a putative MYB family transcription factor (Gene locus name: LOC_Os02g46030.1) was identified. The predicted protein product comprised 491 amino acids with a conversed MYB domain at the N-terminal and was designated* CMYB1*.

**Figure 1:**
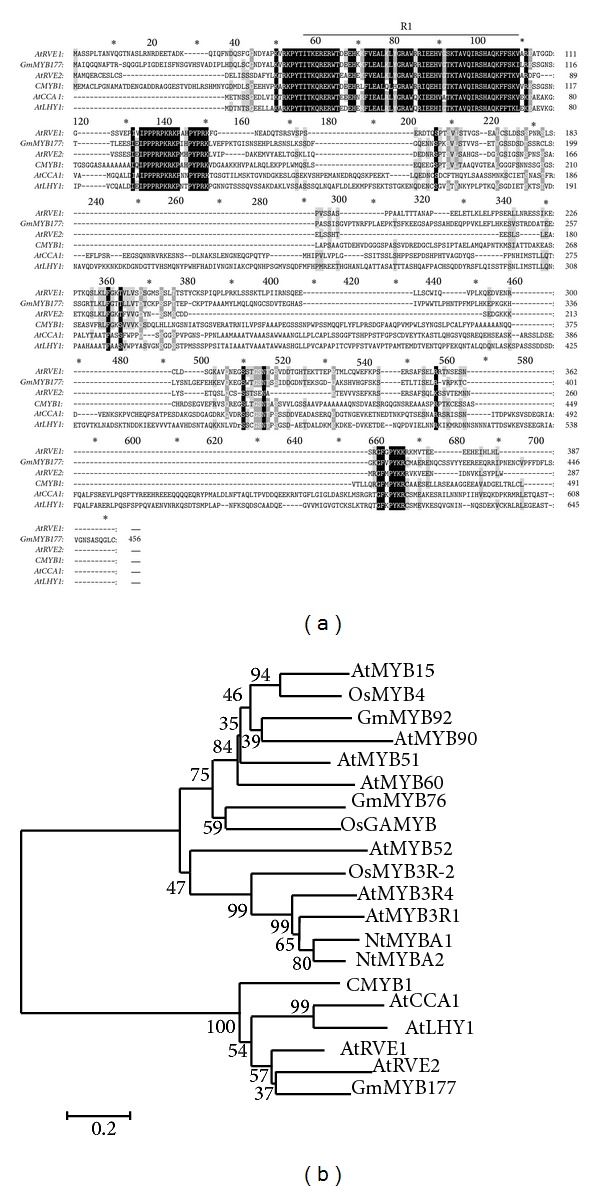
Multiple sequences alignment and phylogenetic tree analysis of amino acid sequences of* CMYB1* with other MYB proteins. (a) Multiple sequences alignment of* CMYB1* with the other R1-type MYB proteins. The upper line indicates the R1 repeat. GenBank accession numbers for the sequences:* AtRVE1* (AT5G17300),* AtRVE2* (AT5G37260),* AtCCA1* (ATU28422),* AtLHY1* (AT1G01060), and* GmMYB177* (DQ822925). (b) Phylogenetic tree analysis of* CMYB1* with other MYB proteins. The neighbor-joining tree was constructed with MEGA 4.0. Branch numbers represent a percentage of the bootstrap values in 1,000 sampling replicates and the scale bar indicates branch length. GenBank accession numbers for the sequences:* AtMYB15* (Y14207),* OsMYB4* (D88620),* GmMYB92* (DQ822903),* AtMYB90* (AT1G66390),* AtMYB51* (AT1G18570),* AtMYB60* (AT1G08810),* GmMYB76* (DQ822895),* OsGAMYB* (AK119607),* AtMYB52* (AT1G17950),* OsMYB3R-2* (BAD81765),* AtMYB3R4* (AT5G11510),* AtMYB3R1* (AF176005),* NtMYBA1* (AB056122), and* NtMYBA2* (AB056123).

*CMYB1* belongs to the R1-type subfamily of MYB proteins and was homologous to many other MYB transcription factors containing single MYB domain (Figure 1(a)). Furthermore, a phylogenetic tree was constructed using neighbor-joining method to investigate the evolutionary relationship among several MYB proteins (Figure 1(b)). The result revealed that* CMYB1* was clustered with AtRVE1 [29], AtRVE2 [30], and GmMYB177 [31], whereas other MYB proteins containing two or three MYB repeats were categorized into another big branch.* GmMYB177* has been reported to confer stress tolerance in transgenic plants [31]. The phylogenetic clustering suggests that* CMYB1* might functionally relate to its orthologues in* Arabidopsis *and soybean.

### 3.2. The Tissue-Specific Expression Pattern of* CMYB1*

**Figure 2:**
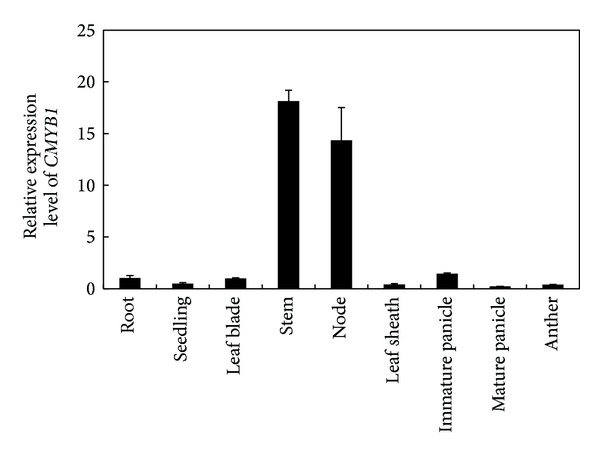
The qRT-PCR analysis of expression of* CMYB1* in various rice tissues.

To explore the tissue-specific gene expression pattern, a total of nine rice tissues at different stages were employed to analyze* CMYB1* expression. Through qRT-PCR assay, it was observed that* CMYB1 *was highly expressed in rice stem and node under normal growth conditions (Figure 2). The stems and nodes of rice plants contain more active cells being proliferating and growing. The MYB proteins have been suggested with the roles in the regulation of cell cycle genes by directly binding their promoter regions. Stress regulated OsMYB3R-2 could directly target* OsCycB1;1*, which was involved in the G2/M phase transition and further regulated cell cycle [19]. Therefore,* CMYB1* probably also plays a role in modulating cell cycle in rice stem and node and involves the internodes elongation.

### 3.3. *CMYB1* Is Induced by Multiple Abiotic Stresses and ABA Treatment

**Figure 3:**
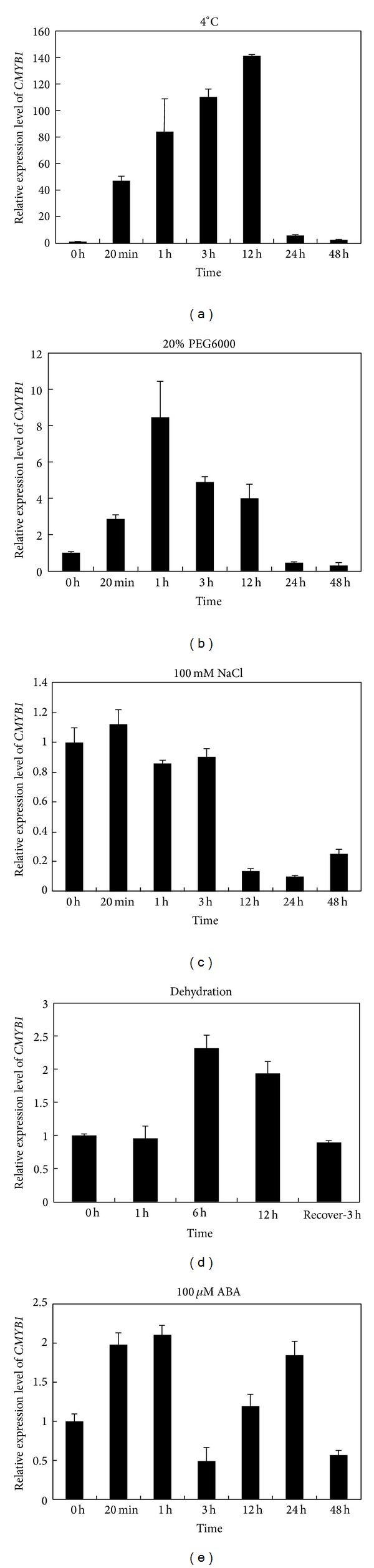
The abiotic-stress-induced expression pattern of* CMYB1*. Jiucaiqing three-leaf seedlings were treated with 4°C (a), 20% PEG6000 (b), 100 mM NaCl (c), dehydration (d), and 0.1 mM ABA (e), respectively.

To verify the transcriptome data, the expression pattern of* CMYB1* in rice seedlings under different stress conditions was investigated. All seedlings were kept under continuous light for 48 h before treatment and the light was continuous during the whole treatments. Consistent with the transcriptome data, the transcription level of* CMYB1 *was induced dramatically under 4°C in a very short time and gradually accumulated up to 12 hour with nearly 140-fold as compared to control (Figure 3(a)). For 20% PEG6000 stress,* CMYB1* was upregulated in 20 minutes and the expression reached to the top in 1 h with about 8-fold as compared to control (Figure 3(b)). However,* CMYB1* was negatively regulated by salt treatment at the later stage of the treatment (Figure 3(c)). For dehydration, the* CMYB1 *was upregulated after 6 h treatment and decreased thereafter. After recovery in nutrition solution, it was found that* CMYB1* expression level decreased to the baseline (Figure 3(d)). The expression of* CMYB1* was also slightly induced by exogenous ABA treatment (Figure 3(e)).

### 3.4. Expression of* CMYB1* Is Involved in Circadian Rhythm

**Figure 4:**
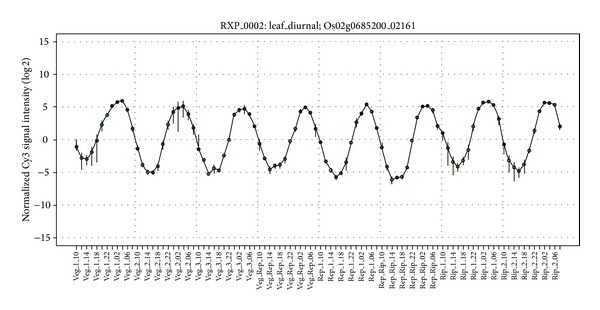
Expression of* CMYB1* in rice response to circadian rhythm in leaves under different developmental stages. Veg.: vegetative; Rep.: reproductive; Rip.: ripening. The gene expression analysis is based on RiceXPro microarray data [24].

Through investigation of* CMYB1 *expression in RiceXPro microarray datasets (http://ricexpro.dna.affrc.go.jp/), it was found that expression of* CMYB1 *was significantly subjected to circadian rhythm regulation. The similar results were obtained in rice leaves during different developmental stages, including vegetative, reproductive, and ripening stages (Figure 4). It seems that* CMYB1* gene was highly expressed in middle night and lowly expressed in middle day in every stage, suggesting that* CMYB1 *might function more specifically in the night. As* CMYB1 *was largely induced upon cold stress, it was hypothesized that* CMYB1* probably functions in regulating expression of genes involved in cold tolerance in the night to maintain plants normal growth, and its expression is downregulated in the day with the increased temperature. Interestingly, two other orthologues of* CMYB1, REVEILLE1* (*RVE1*) and* RVE2*, were also involved in circadian rhythm. Although inactivation of RVE1 does not affect circadian rhythmicity, it was speculated that* RVE1 *was a clock output affecting plant development. It was also suggested that* RVE2 *was possibly part of a regulatory feedback loop that controls a subset of the circadian outputs. As* CMYB1* expression was also strictly circadian rhythm regulated, this branch of MYB family might be involved in circadian rhythmicity, especially functioning in the regulation of circadian outputs regulating plant development and adaptation to environment changes.

### 3.5. cis-Acting Elements Present in* CMYB1* Promoter Region 

The promoter sequence of* CMYB1 *was analyzed through the MatInspector program (http://www.genomatix.de/). The result showed that the promoter sequence of* CMYB1* contained many putative plant hormone-related* cis*-acting elements, such as ABA-responsive element (ABRE), auxin response element (AuxRE), brassinosteroid (BR) response element (BRRE), and ethylene response element factors (EREF), indicating that* CMYB1 *might be a central crosstalk of various plant hormones regulating plant growth and development. The presence of ABRE in the promoter region and the fact that* CMYB1* was induced by exogenous ABA treatment suggested that* CMYB1* probably functions in the ABA-dependent pathway of stress response in rice. Interestingly, we also found a circadian-related* cis-*element existing in the promoter region (Table 1), indicating that regulation of* CMYB1* by circadian rhythm might be mediated by this element.

### 3.6. *CMYB1* Is Localized in the Nuclei

**Figure 5:**
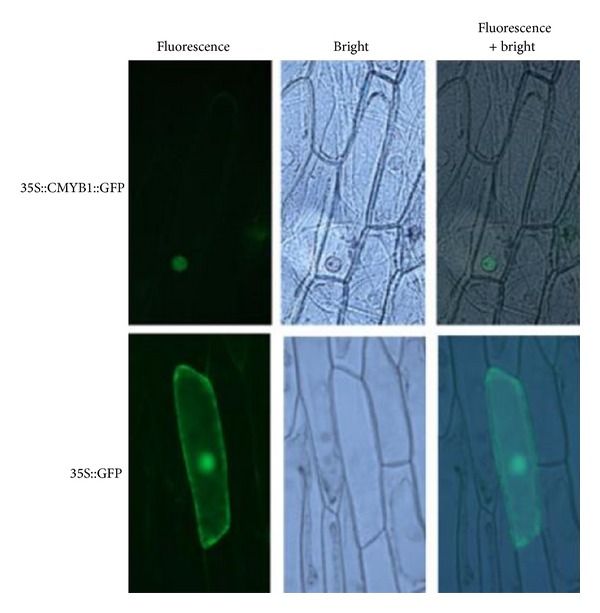
Subcellular localization of* CMYB1*. The 35S::*CMYB1*::GFP which was constructed in pA7 vector and the empty vector harboring 35S::GFP were transformed into onion epidermis cells, respectively.

To investigate the subcellular location of* CMYB1*, the coding regions of the whole* CMYB1* cDNA were fused with GFP gene, and the resulting construct was introduced into onion epidermal cells by particle bombardment. Localization of fusion protein was determined by visualization with fluorescence microscopy. The 35S::*CMYB1*::GFP fusion protein was localized to the nuclei of onion epidermal cell (Figure 5). By contrast, the control 35S::GFP fusion protein without* CMYB1* was distributed throughout the whole cell, indicating that* CMYB1* was nuclear-localized protein. Therefore,* CMYB1* has a typical feature of a transcription factor that localizes in the nucleus of a cell.

### 3.7. *CMYB1* Functions as a Transcriptional Activator

**Figure 6:**
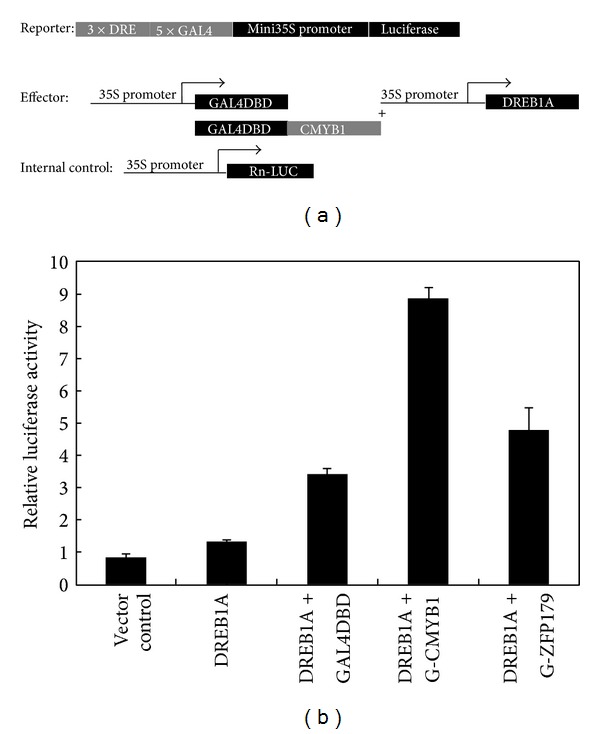
Transcriptional activity analysis of* CMYB1* in rice protoplast via dual luciferase reporter assay system. (a) Construction of reporter, effector, and internal control vectors. (b) The luciferase activities. Vector control contained reporter and internal control plasmids; DREB1A contained reporter, internal control, and 35S::DREB1A protein plasmids; DREB1A+GAL4DBD contained reporter, internal control, 35S::DREB1A protein, and 35S::GAL4DBD protein plasmids; DREB1A+G-*CMYB1* contained reporter, internal control, 35S::DREB1A protein, and 35S::GAL4DBD-*CMYB1* fusion protein plasmids; DREB1A+G-ZFP179 contained reporter, internal control, 35S::DREB1A protein, and 35S::GAL4DBD-ZFP179 fusion protein plasmids.

The transcriptional activity of* CMYB1* was examined via dual luciferase reporter assay system in rice protoplast. Protoplasts were transformed with effector plasmids and reporter plasmid containing GAL4, DRE fragments, and minimal cauliflower mosaic virus (CaMV) 35S promoter in upstream of luciferase. The effector plasmids were composed of two parts. One was 35S::DREB1A expression protein that could bind to the DRE fragments. Another one was 35S promoter fused to the GAL4 DBD (DNA binding domain) or GAL4DBD-*CMYB1* cDNA. 35S promoter fused to the GAL4DBD-ZFP179 cDNA was used as positive control (Figure 6(a)). As shown in Figure 6(b), coexpression of DREB1A and GAL4DBD proteins in protoplasts showed induction in the expression of the luciferase reporter gene as compared to the expression of DREB1A alone. Compared with GAL4 DBD protein, coexpression of DREB1A and GAL4DBD-*CMYB1* displayed significant induction in the expression of the luciferase reporter gene, which was about 3-fold to the one harboring DREB1A and GAL4DBD proteins. This result indicated that* CMYB1* had transcriptional activity in rice protoplast and functioned as a transcriptional activator. Based on the subcellular localization and transcription activation activity, it could be confirmed that* CMYB1* functions as a transcription activator in rice cells.

## 4. Conclusion

In this paper, we reported a novel MYB transcription factor gene* CMYB1 *which was dramatically induced by cold stress.* CMYB1* is a nuclear protein exhibiting transcriptional activation activity, implying that* CMYB1* functions as a transcription activator in rice cells. Interestingly, we found that expression of* CMYB1* was significantly regulated by circadian rhythm. As* CMYB1 *was largely induced upon cold stress, it is hypothesized that* CMYB1* probably promotes cold-responsive gene expression to respond to low temperature in the night. With temperature increases in the day, expression of* CMYB1 *gradually decreases. However, this hypothesis needs further experimental validation by use of* CMYB1* null mutant or transgenic approach.

## Figures and Tables

**Table 1:** Predicted *cis-*elements in the promoter region of *CMYB1*.

Element name	Element sequence^a^	Position^b^
ABA response elements (ABRE)	tctgatgaCGTGgaccg	−1441–−1425
	tggcctgaCGTGtcaaa	−988–−972
	cacgataaGGTGgccct	−688–−652
	gagtcctACGTggcgcg	−264–−248

Auxin response element (AuxRE)	ttaTGTCcccgtc	−1422–−1410

Brassinosteroid response element (BRRE)	cctgaCGTGtcaaaaaa	−985–−969
	gcgctCGTGtggacggg	−914–−898

Ethylene response element factors (ERE)	aGCGAaagtcccaaagtcc	−964–−946

GCC box family (GCC)	gagtccCGCCatg	−1270–−1258

Heat shock element (HSE)	gcgattTTTCgtgaatt	−1205–−1189
	gaaaaaaataaAGAAaa	−368–−352

NAC transcription factor recognition site	ttctttactccccgcCACGtcatcccc	−1368–−1342
	tagtgcgacggggccTACGtaacgctc	−205–−179

Salt/drought responsive element	gttcgGTGGtggatt	−1039–−1025

Circadian LELHC	catCAAcaacATCgag	−123–−108

^a^The capital letters represented the core sequence in the predicted *cis*-element.

^b^Position displayed the nucleotide position relative to the up-stream of ATG of *CMYB1*.
